# Impact of Auditory Experience on the Structural Plasticity of the AIS in the Mouse Brainstem Throughout the Lifespan

**DOI:** 10.3389/fncel.2019.00456

**Published:** 2019-10-15

**Authors:** Eun Jung Kim, Chenling Feng, Fidel Santamaria, Jun Hee Kim

**Affiliations:** ^1^The Department of Cellular and Integrative Physiology, UT Health San Antonio, San Antonio, TX, United States; ^2^The Department of Biology, University of Texas, San Antonio, TX, United States

**Keywords:** axon initial segment, auditory brainstem, MNTB, auditory experience, mouse

## Abstract

Sound input critically influences the development and maintenance of neuronal circuits in the mammalian brain throughout life. We investigate the structural and functional plasticity of auditory neurons in response to various auditory experiences during development, adulthood, and aging. Using electrophysiology, computer simulation, and immunohistochemistry, we study the structural plasticity of the axon initial segment (AIS) in the medial nucleus of the trapezoid body (MNTB) from the auditory brainstem of the mice (either sex), in different ages and auditory environments. The structure and spatial location of the AIS of MNTB neurons depend on their functional topographic location along the tonotopic axis, aligning high- to low-frequency sound-responding neurons (HF or LF neurons). HF neurons dramatically undergo structural remodeling of the AIS throughout life. The AIS progressively shortens during development, is stabilized in adulthood, and becomes longer in aging. Sound inputs are critically associated with setting and maintaining AIS plasticity and tonotopy at various ages. Sound stimulation increases the excitability of auditory neurons. Computer simulation shows that modification of the AIS length, location, and diameter can affect firing properties of MNTB neurons in the developing brainstem. The adaptive capability of axonal structure in response to various auditory experiences at different ages suggests that sound input is important for the development and maintenance of the structural and functional properties of the auditory brain throughout life.

## Introduction

Continuous auditory input is necessary for the proper development and maintenance of the auditory system. Peripheral hearing deficits can occur during development (congenital deafness) and through aging (presbycusis), lead to reduced auditory input, and generate a reorganization of central auditory circuits (Butler and Lomber, 2013; Lin et al., 2014). Alterations in the structure and function of the central auditory system impair auditory processing even after peripheral sound sensitivity is restored, such as with hearing aids (Sweetow, 2005; Atcherson et al., 2015). To restore proper circuit processing, it is essential to understand how sound-evoked activity refines and modulates the structural plasticity, and thereby the functional connectivity of the central auditory system. In animal models, sound deprivation in congenital deafness or by surgical ablation of the cochlea resulted in anatomical and functional changes in subcortical nuclei (Russell and Moore, 1995; Grande et al., 2014). In the auditory brainstem, congenital deafness is associated with a reduction in the number and size of neurons in the cochlear nucleus (Hashisaki and Rubel, 1989; Saada et al., 1996), decreased inhibitory inputs on medial superior olivary neurons (Kapfer et al., 2002; Tirko and Ryugo, 2012), and disruption of the tonotopic organization in the MNTB (von Hehn et al., 2004; Leao et al., 2006). Although the structural consequences of congenital and early-onset deafness have been studied in several animal models, the physiological consequences or homeostatic adaptation processes in the central auditory system in response to sound experiences throughout life are not yet determined.

As the key axonal domain for generating action potentials (APs), the AIS determines neuronal excitability, modulates neuronal output, and controls auditory processing along the ascending auditory pathway (Kuba et al., 2006; Gründemann and Häusser, 2010). In the chick brainstem, structural properties of the AIS depend on its tonotopic location - the spatial arrangement of where sounds of different frequencies are processed in the brain (Kuba et al., 2006, 2014). The AIS of chick neurons elongates to increase excitability when synaptic inputs are removed by cochlear ablation, suggesting a contribution of the AIS to the homeostatic control of neural activity (Kuba, 2012). In cultured hippocampal neurons, the AIS is located farther from the soma when neuronal activity is increased (Grubb and Burrone, 2010). When the AIS is located proximal to the soma, APs are easier to initiate, thus increasing neuronal excitability, whereas a more distal location reduces neuronal excitability (Grubb and Burrone, 2010; Kole and Stuart, 2012). Thus, adaptive changes in AIS structure and position have been thought to alter intrinsic excitability in a homeostatic direction (Kole and Stuart, 2012; Wefelmeyer et al., 2016). However, depending on somatodendritic morphology in various neurons, the distal shift of the AIS location theoretically reduces the threshold and rheobase of AP and promotes AP generation (Gulledge and Bravo, 2016; Kole and Brette, 2018). Importantly, the structural properties and plasticity of the AIS vary among individual neurons across brain regions (Chand et al., 2015; Höfflin et al., 2017). Although the structural plasticity of the AIS has been studied in the chick brainstem, concerning the heterogeneity of AIS structure across species, it is important to explore AIS plasticity in response to auditory input within a mammalian system. Understanding how *in vivo* auditory experience alters the AIS of auditory neurons throughout the lifespan of the mammalian brain from development to aging is critically important for developing targeted strategies to remedy potential long-term deficits of the auditory brain at specific ages.

Here, we investigated the spatial distribution and location of the AIS in the mouse auditory brainstem at different ages, focusing on AIS length, position, and tonotopic differentiation. Sound modifications alter the structural properties of AIS and affect tonotopy in the auditory brainstem. Electrophysiological and computer modeling studies indicate that there is a relationship between AIS structural plasticity and neuronal excitability in the MNTB. The results demonstrate that structural plasticity of the AIS in auditory neurons is influenced by sound-evoked activity.

## Materials and Methods

### Animals

Either sex of C57BL/6 mice and whirler mice with a C57BL/6 background were used in accordance with the guidelines approved by the University of Texas Health Science Center, San Antonio (UTHSCSA) Institutional Animal Care and Use Committee protocols. All mice were housed in the institutional animal facilities on a 12-h light/dark cycle.

### Sound Modification in Mice

Mice in the sound stimulation group were given acoustic stimulation with 80dB single tones of 16 kHz for 7 days, 3 h per day from P13 to P19 (in postnatal development) or from P38 to P44 (in early adulthood) in a sound attenuation chamber (Med Associates, Albans, VT). Acoustic stimuli were generated by an auditory evoked potentials workstation [Tucker-Davis Technologies (TDT), Alachua, FL]. The signals consisted of a series of amplitude-modulated square waves (duration 0.1 ms, repeat rate 16/s) through TDT multifield magnetic speakers. Whirler mice, a model of congenital deafness (Xu et al., 2017), were used for the sound deprivation model and were provided by Dr. M. A. Bhat’s laboratory (UTHSCSA). As a model of hearing loss during adulthood, mice (at P70) were exposed to high-pressure air (13 psi shock wave, ∼183 dB) generated by a large (17-inch diameter) compressed-air shock tube (Cho et al., 2013; Choi et al., 2015). ABR test and immunostaining were performed in 1 week after the blast exposure (at P80).

### Slice Preparation

After rapid decapitation, the brains were quickly removed from the skull and immediately immersed in ice-cold low-calcium artificial cerebrospinal fluid (aCSF) containing (in mM): 125 NaCl, 2.5 KCl, 3 MgCl2, 0.1 CaCl2, 25 glucose, 25 NaHCO3, 1.25 NaH2PO4, pH 7.3–7.4 bubbled with carbogen (95% O2, 5% CO2; osmolarity of 310–320 mOsm). For c-fos staining, animal was sacrificed within 1 h after sound stimulation. Then, transverse 200-μm-thick brainstem slices containing the MNTB were collected using a Vibratome (VT1200S, Leica, Germany). Collected slices were prepared for electrophysiology or immunohistochemistry experiments.

### Electrophysiology

After vibratome sectioning, slices were further incubated in a chamber containing normal aCSF bubbled with carbogen at 35°C for 30 min and then were kept at room temperature. The normal aCSF was the same as the low-calcium aCSF, except that 3 mM MgCl2 and 0.1 mM CaCl2 were replaced with 1 mM MgCl2 and 2 mM CaCl2. Whole-cell patch-clamp recording was carried out on postsynaptic principal neurons in the MNTB nuclei at room temperature (∼24°C). Action potentials (APs) were recorded in normal aCSF using the voltage or current-clamp mode of the EPC-10 (HEKA Electronik, Lambrecht/Pfalz, Germany). The pipettes were filled with an internal solution containing (in mM) 125 K-gluconate, 20 KCl, 5 Na2-phosphocreatine, 10 HEPES, 4 Mg-ATP, 0.2 EGTA, and 0.3 GTP, pH adjusted to 7.3 with KOH. The holding potential was −70 mV in the voltage-clamp mode. Current-clamp protocols were 200 ms in duration with current steps from −50 to 250 pA (50 pA increments). Patch electrodes had resistances of 4–5 M Ω. Series resistance was < 20 M Ω, with 80% compensation. In our solution composition (extracellular K^+^ concentration of 2.5 mM and intracellular K^+^ concentration of 150 mM), the calculated equilibrium potential for potassium is around −106 mV, and liquid junction potential is ∼20 mV. Membrane potentials were not corrected for this constant liquid junction potential between the extracellular and pipette solution. In the current clamp recordings, the baseline potential was around −70 mV with ∼−20 pA injection, and the amplitude of action potential was defined as the potential between the baseline potential to the peak. The threshold of action potential (AP) was determined by the point where dV/dt exceeds 10V/s and the amplitude of AP from this threshold to the AP peak in the plot of dV/dt and voltage. Data were analyzed and displayed with Igor Pro (Wavemetrics, Lake Oswego, OR, United States).

### Immunohistochemistry

Brainstem slices were fixed with 4% (w/v) paraformaldehyde in phosphate-buffered saline (PBS) for 20 min and were washed with PBS three times. Free-floating slices were blocked in 4% goat serum and 0.3% (w/v) Triton X-100, 0.1% Tween 20 in PBS for a 1 h and then were incubated with primary antibody overnight at 4°C. The following primary antibodies were used: rabbit anti β4 spectrin (1:250) as previously described (Saifetiarova et al., 2018), mouse monoclonal anti-MAP2 (1:200; Millipore Cat# MAB3418, RRID:AB94856), mouse monoclonal anti-ankyrinG (AnkG; 1:100; UC Davis/NIH NeuroMab Facility Cat# 75-146, RRID:AB_10673030), mouse monoclonal anti-Kv1.2 (1:250; UC Davis/NIH NeuroMab Facility Cat# 75-008, RRID:AB_10673030), mouse monoclonal anti-Na-pan (1:100; Sigma-Aldrich Cat# S8809, RRID:AB_10673030), and rabbit polyclonal anti-c-Fos (1:100; Synaptic System Cat# 226003). After 3 washes with PBS containing 0.1% Tween 20, slices were incubated with different Alexa-488 goat anti-mouse IgG1 or 568 goat anti-mouse IgG2b or 568 goat anti-rabbit or 647 goat anti-guinea pig secondary antibodies (1:1000; Invitrogen) accordingly for 2 h at room temperature. Slices were then rinsed with PBS containing 0.1% Tween 20 and were coverslipped using the mounting medium (Vectashield; Vector Laboratories). Stained slices were viewed on a confocal laser-scanning microscope (Carl Zeiss LSM-710) at 488, 568, and 633 nm using a pinhole of 1 AU and 20x (0.8 NA), 40x (oil-immersion, 1.30 NA) objectives. Z-stack images were acquired at a digital size of 1,024 × 1,024 pixels with optical section separation (*z* interval) of 0.5 um. The images were imported into Fiji (Image J, Schindelin et al., 2012), ZEN (Carl Zeiss) and Amira software (FEI, Netherlands) for the analysis. To analyze AIS structure, either the 2D compressed or the 3D reconstructed Z-stack confocal images of MNTB neurons were utilized. Most analyses were performed using the 2D compressed images because there was no significant difference between the results from the 2D- and 3D- analysis (the AIS from normal mice, 16.89 ± 0.245, *n* = 18 for 2D vs. 17.17 ± 0.36, *n* = 18 for 3D, *p* = 0.5245). The AIS was determined as the axonal domain where the fluorescence intensity of β4 spectrin was > 10% of the peak signal. We did not perform background subtraction of images, because the signal of β4 spectrin is very specific to axonal domains including the AIS. For the analysis of AIS structure, we utilized a line profile of AIS from the end of distal edge of the AIS, which was defined by a paranodal protein, caspr, to the end of the proximal edge of the AIS, where the signal of β4 spectrin was < 10% of the peak signal. 3D image (Amira) confirmed the AIS structure including length, volume, and diameter obtained from 2D images by Fiji or ZEN software. For analyzing the AIS with a strong curvature, we utilized the segmental line profile in the ImageJ. For c-fos analysis, the double staining against MAP2 and c-Fos were performed from normal hearing group (P20) and sound stimulation group (P20) simultaneously to exclude the difference of fluorescence intensity. MAP2- and c-fos-positive cells were counted using cell counter plugin of Fiji software. Only the cells with c-fos-positive nucleus were counted and the constant threshold level of fluorescence intensity was used in each slices. The percentage of c-fos-positive cells were calculated by dividing the number of MAP2 -positive MNTB neurons in each slices.

### *In vivo* Auditory Brain Stem Response (ABR) Test

ABR recordings were conducted as described previously (Kim et al., 2013). Briefly, mice were anesthetized with 4% isoflurane and maintained with 2% isoflurane during recording (1 l/min O2 flow rate). During ABR recordings, the body temperature was maintained between 35 and 37°C using a non-electric heating pad and equally controlled in different experimental groups. ABR recordings were performed in a sound attenuation chamber (Med Associates, Albans, VT). Subdermal needle electrodes (Rochester Electro-Medical, Lutz, FL, United States) were placed on the top of the head, ipsilateral mastoid, and contralateral mastoid as the active, reference, and ground electrodes, respectively. The signal differences in the ABRs between the vertex and the mastoid electrodes were amplified and filtered (100–5,000 Hz). Acoustic stimuli were generated by an auditory evoked potentials workstation [Tucker-Davis Technologies (TDT), Alachua, FL, United States]. Closed-field click stimuli were presented to the left ear. The signals consisted of a series of amplitude-modulated square waves (duration 0.1 ms, repeat rate 16/s) through TDT multifield magnetic speakers. The sound stimuli were delivered through a 10-cm plastic tube (Tygon; 3.2-mm outer diameter) at a repeat rate of 16/s. Sound intensities ranged from 90 to 20 dB, with 5-dB decrements, and responses to 512 sweeps were averaged.

### Computer Simulation

A compartmental model of an MNTB cell was implemented based on previous publications (Leão et al., 2008; Wang et al., 1998). The structure of the cell consisted of one primary dendrite with a length of 40 μm and a uniform diameter of 3 μm. The dendrite was connected to a spherical soma with a diameter of 30 μm. The axonal segment was divided between the hillock and the initial segment (AIS), both with a diameter of 2 μm. The length of the hillock was 16 μm and the length of the AIS was 13 μm (Figure 3). The model had active conductance. The density and kinetics are described in the following Tables 1,  2.

**TABLE 1:** Conductance and distribution of ion channels.

**Conductance (S/cm^2)**	**Dendrite**	**Soma**	**Hillock**	**AIS**
Leak	0.1055e-3	0.1055e-3	0.1055e-3	0.1055e-3
Na_V_1.1				385e-3
K_V_3.1		186.4623e-3		
K_V_1.1				2.95e-3
H channel	0.0414e-3	0.0638e-3		

**TABLE 2:** Kinetics of active conductance.

**Name**	**State**	**N**	**Model**	
Na_V_1.1	Activation	3	α_m_ = 76.4*e*^0,037*v*^	α_h_ = 0.00013*e*^−0.1216*v*^
	Inactivation	1	β_m_ = 6.930852*e*^−0.043*v*^	β_h_ = 1.999*e*^0.0384*v*^
K_V_1.1	Activation	1	α_*l*_ = 1.2*e*^0.03512*v*^	α_*r*_ = 0.0438*e*^−0.0053*v*^
	Inactivation	1	β_*l*_ = 0.2248*e*^−0.0319*v*^	β_*r*_ = 0.0562*e*^−0.0047*v*^
K_V_3.1	Activation	1	α_*n*_ = 0.2719*e*^0.04*v*^	α_*p*_ = 0.0073*e*^−0.1942*v*^
	Inactivation	1	β_*n*_ = 0.1974*e*^0*v*^	β_*p*_ = 0.0936*e*^−0.0058*v*^
			${\text{i}}_{\text{k}}=\,\bar{g}\,n^{3}\,\left(1-\gamma +\gamma \,p\right)\,\left(v-e_{k}\right)$	
			γ = 0.1	
H channel	Activation	1	$\alpha =\frac{0.63}{1000}\,e^{-0.063\,\left(v+73.1\right)}$	
			$\beta =\frac{0.63}{1000}\,e^{0.078\,\left(v+73.2\right)}$	

Simulations consisted of injecting constant current to the soma and quantifying the shape and number of spikes generated over a period of 700 ms. The model was implemented in Python-NEURON (ver 7.3) and analyzed with custom routines in Matlab (Mathworks, Natick, MA, United States).

### Statistical Analysis

All statistical analyses were performed in Prism (GraphPad Software). Normality of datasets was analyzed using the D’Agostino and Pearson’s omnibus test. Parametric or non-parametric tests were carried out accordingly. α values were set to 0.05, and all comparisons were two-tailed. To compare two groups, unpaired *t*-test or Mann–Whitney *U* test was carried out, respectively. For three or more groups, the Kruskal–Wallis test with Dunn’s *post hoc* test or one-way ANOVA with Turkey’s multiple comparison test was used. For the comparison of the AIS of HF and LF MNTB neurons from three groups, we analyzed the mean value from individual animal as the experimental unit, and utilized 2-way ANOVA with Sidak’s multiple comparison test. The significance was determined at *P*-values < 0.05. To analyze the tonotopy of AIS structure, we analyzed the mean value from individual animal as the experimental unit using 2-way ANOVA with Sidak’s multiple comparison test. Data were shown as the mean ± standard error of the mean (s.e.m.) with *n* values representing the number of animals per experimental group or the number of neurons per group where indicated. In box and whisker plot, boxes indicate 25–75% interquartile range and horizontal lines in boxes indicate the median. Whiskers show 5% ∼ 95% range and dots show outliers that reside outside the whisker range.

## Results

### The AIS of Auditory Neurons Are Differentiated by Structure and Function Along the Tonotopic Axis in the Mouse MNTB

**FIGURE 1:**
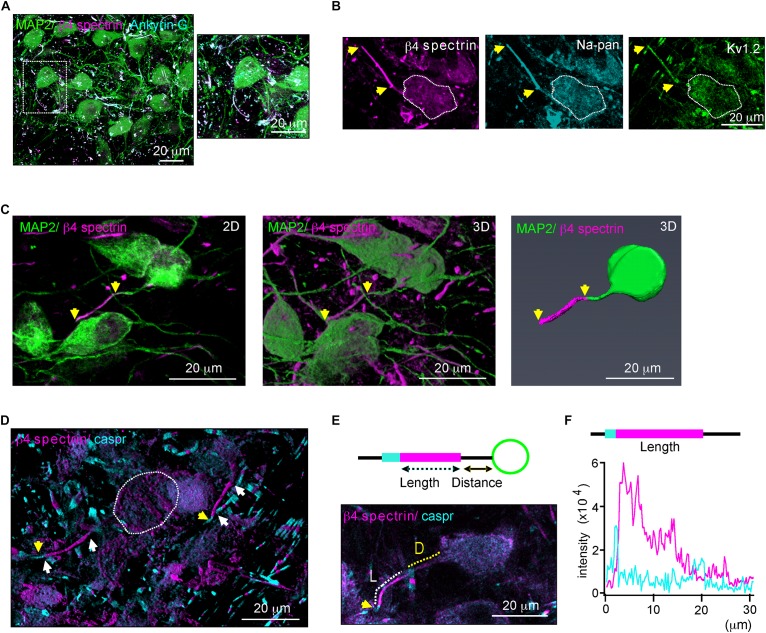
β4 spectrin is co-localized with ankyrinG and Na^+^ and K^+^ channels. **(A)** Immunostaining of MNTB neurons with MAP2 (blue), β4 spectrin (green) with ankyrinG (magenta) of P20 normal hearing mice. Dotted line indicates the soma of MNTB neuron. **(B)** MNTB neurons immunostained with β4 spectrin (cyan), voltage-activated Na^+^ channel (Na-pan, green) and K^+^ channel (K_V_1.2, magenta). Dotted line indicates the soma of MNTB neuron. **(C)** 2D compressed Z-stack images (left) and 3D reconstructed Z-stack images using Zen (middle) and Amir (right) of the same MNTB neuron immunostained with MAP2, β4 spectrin. Yellow arrows indicate AIS length. **(D)** Immunostaining of the MNTB with β4 spectrin and caspr. White arrows indicate the AIS proximal and distal ends. Capsr (magenta, yellow arrows) was located at the next of the AIS distal end. **(E)** (Top) Diagram of the MNTB neuron with soma (green), AIS (cyan), and paranode (magenta) shows the AIS length and distance from the soma. (Bottom) MNTB neurons immunotained with MAP2 (green), β4 spectrin (magenta) and caspr (cyan). A dotted line indicates the length (L, white) and distance (D, yellow) of AIS structure. **(F)** The intensity profiles of β4 spectrin (magenta) and caspr (cyan) immunostaining along the AIS of MNTB neuron indicated by length and distance in **E**.

We characterized structural properties of the AIS in the mouse MNTB (P20) using β4 spectrin immunostaining, which was present along the AIS and co-localized with ankyrin G, represented the AIS of MNTB neurons (Figure 1A). β4 spectrin was also co-localized with voltage-activated Na^+^ and K^+^ channels at the AIS of MNTB neurons (Figure 1B). To analyze AIS structure, either the 2D compressed or the 3D reconstructed Z-stack confocal images of MNTB neurons were utilized (Figure 1C). We selected the AIS with the distinct distal end, which was defined by a paradonal protein, Caspr (Xu et al., 2017), and with the proximal end, where β4 spectrin intensity was < 10% of the peak intensity, for the analysis of AIS structure (Figure 1D). To quantify AIS length and position, we measured the length of the region, where the intensity of β4 spectrin was > 10% of the peak signal, and the distance from soma to the proximal end of the AIS (Figures 1E,F).

**FIGURE 2:**
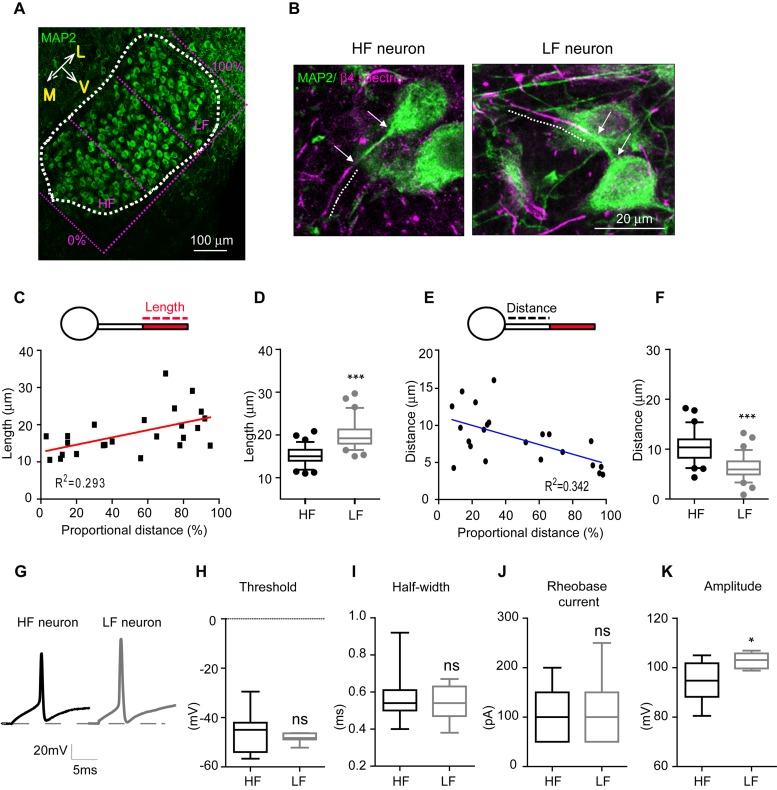
AIS length and position of MNTB neurons depend on their spatial location along the tonotopic axis. **(A)** Immunostaining of MNTB neurons with MAP2 along the tonotopic axis. The MNTB was proportionally divided by the distance from the medial edge (0%) to the lateral edge (100%). HF neurons were located in < 30%, and LF neurons were located in > 70% of the proportional distance. **(B)** Representative HF and LF neurons immunostained with MAP2 and β4 spectrin from normal hearing mice at P20. A dotted line shows AIS length and white arrows point the distance between the AIS and the soma. **(C)** Plot of AIS length of MNTB neurons against their location (0–30%: HF neurons, 70–100%: LF neurons). **(D)** Box and whisker plot of AIS length in HF (black) and LF (gray) neurons of the MNTB at P20. Boxes indicate 25–75% interquartile range and horizontal lines in boxes indicate the median. Whiskers show 5%–95% range and dots show outliers that reside outside the whisker range. **(E)** The plot of AIS distance of MNTB neurons against their location (0–30%: HF neurons, 70–100%: LF neurons). **(F)** Box and whisker plot of AIS distance in HF (black) and LF (gray) neurons of the MNTB at P20. **(G)** APs of HF (black) and LF neurons (gray) evoked by step-like current injection (100 pA). **(H–K)** Summary of the threshold, half-width, rheobase current and amplitude of APs. ^∗^*p* < 0.05, ^∗∗∗^*p* < 0.0001, Mann–Whitney *U* test.

To quantify AIS length and location along the medial-lateral axis in the MNTB from individual mouse brainstems (*n* = 6 mice at P20), the MNTB was proportionally defined by the percent of the total distance from medial to lateral edges of the MNTB, where 0% represents the medial edge and 100% represents the lateral edge (Figure 2A). High-frequency sound responding neurons (HF neurons) were defined as neurons located within 0–30% of the total distance from medial to lateral edges, and low-frequency responding neurons (LF neurons) were defined as neurons located within 70–100% of the distance from the medial to the lateral edge (Figures 2A,B). AIS length was gradually increased from medial to lateral MNTB neurons. Plotting AIS length against the proportional distance showed the distribution of AIS length as estimated by linear regression analysis (*R*^2^ = 0.293, *n* = 24 cells, *p* = 0.0063; Figure 2C). AIS length was significantly shorter in HF neurons than LF neurons. AIS length in HF and LF neurons was 15.1 ± 0.23 μm (*n* = 79 cells) and 19.9 ± 0.37 μm (*n* = 64 cells), respectively (*p* < 0.0001, Mann–Whitney *U* test; Figure 2D). The spatial location of the AIS was quantified by the distance between the proximal end of the AIS and the soma, which was gradually decreased along the medial-lateral axis with a linear correlation (*R*^2^ = 0.342, *n* = 22 cells, *p* = 0.0042; Figure 2E). The AIS of HF neurons were located more distally from the soma than the AIS of LF neurons, which were located proximal to the soma. Thus, AIS distance was significantly longer in HF than LF neurons (10.4 ± 0.34 μm, *n* = 63 cells in HF neurons vs. 6.2 ± 0.24 μm, *n* = 74 cells in LF neurons, *p* < 0.0001, Mann–Whitney *U*-test; Figure 2F).

To examine how structural differentiation of the AIS along the tonotopic map influences intrinsic properties of MNTB neurons, we recorded action potentials from HF (*n* = 11 cells from 5 mice) and LF neurons (*n* = 7 cells from 3 mice), which were evoked by step-current injections in whole-cell recording (Figure 2G). There was no difference in the threshold and half-width of APs, and the rheobase current (threshold: -45.3 ± 2.53 mV in HF neurons vs. -48.3 ± 0.74 mV in LF neurons, *p* = 0.3189; half-width: 0.5 ± 0.04 ms in HF neurons vs. 0.5 ± 0.03 ms in LF neurons, *p* = 0.8917, the rheobase current: 106.5 ± 11.47 pA in HF neurons vs. 107.1 ± 27.66 pA in LF neurons, *p* = 0.8573, Mann–Whitney *U* test; Figures 2H–J). However, HF neurons displayed smaller APs with amplitudes of 94.1 ± 2.66 mV (*n* = 10 cells), while APs from LF neurons had larger amplitudes of 102.8 ± 1.12 mV (*n* = 7 cells, *p* = 0.0250, Mann–Whitney *U*-test; Figure 2K). Taken together, HF neurons with a shorter AIS distally located from the soma displayed a smaller AP, whereas LF neurons with a longer AIS proximally located from the soma exhibited a larger AP in the MNTB. This indicates that MNTB neurons have structural and functional differentiation of the AIS along the tonotopic axis, which is associated with differentiation of their firing properties.

### AIS Tonotopy Is Initiated After Hearing Onset, Progressively Developed, and Stabilized in Adulthood

**FIGURE 3:**
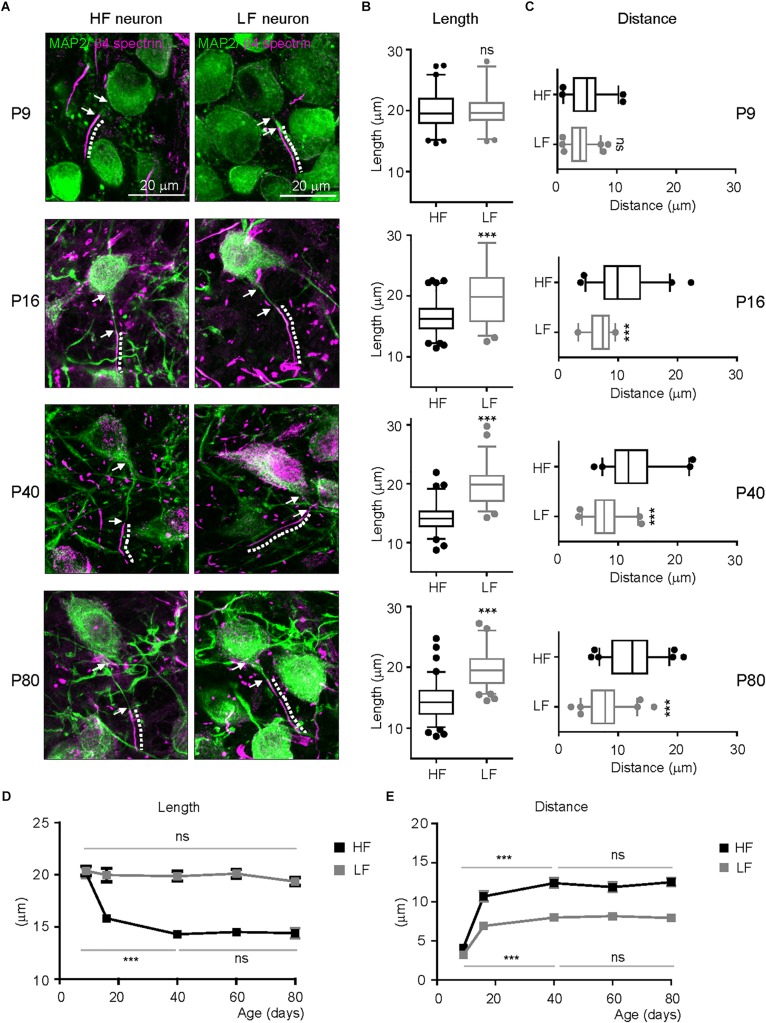
Developmental refinement of AIS structure and tonotopy in the MNTB. **(A)** Representative images of HF and LF neurons, which were immunostained with MAP2 (green) and β4spectrin (magenta) at P9, P16, P40, and P80. The dotted line and white arrow indicate AIS length and distance, respectively. **(B,C)** Summary of AIS length and location in HF (black) and LF (gray) neurons of the MNTB at P9, 16, 40, and 80. Note, the significant difference in AIS length and location between HF and LF neurons appears at P16 after hearing onset. ^∗∗∗^*P* < 0.0001, Mann–Whitney *U* test or unpaired *t*-test. **(D,E)** The plot of AIS length and distance of HF (black) and LF neurons (gray) along postnatal ages (from P9 to P80). ^∗∗∗^*P* < 0.001, Kruskal–Wallis test with Dunn’s *post hoc* test.

In hearing mammals, a rough tonotopy is established before hearing onset, then later refined by sound-evoked activity, generating the sophisticated tonotopy of the mature system (Kandler et al., 2009). To determine when tonotopic differentiation of AIS structure initiates in the mouse auditory brainstem, we examined AIS length and location throughout postnatal development into early adulthood (P9: *n* = 4 mice, P16: *n* = 3 mice, P40: 4 mice, P60: 3 mice and P80: 4 mice). At P9 (before hearing onset at P12), the AIS was distinctly detectable and its length was similar in HF and LF neurons (20.2 ± 0.40 mm, *n* = 61 cells in HF neurons vs. 20.1 ± 0.39 mm, *n* = 47 cells in LF neurons, *p* = 0.8867, Mann–Whitney *U* test; Figures 3A,B). The AIS from both groups was closely located to the soma, and the distance between the proximal end and the soma was 3.8 ± 0.24 mm (*n* = 61 cells) in HF neurons and 4.7 ± 0.37 mm (*n* = 45 cells) in LF neurons (*p* = 0.0505, Mann–Whitney *U* test; Figures 3A,C). There was no significant difference in AIS length and location between HF and LF neurons at P9. At P16 after hearing onset, a significant difference in AIS length and location between HF and LF neurons was observed. The AIS of HF neurons was significantly shorter than the AIS of LF neurons (16.3 ± 0.30 mm, *n* = 84 cells in HF neurons vs. 20.2 ± 0.58 mm, *n* = 59 cells in LF neurons, *p* < 0.0001, unpaired *t*-test; Figures 3A,B). In parallel with changes in length, the AIS of HF neurons moved further away from the soma during postnatal development, resulting in a significant difference between HF and LF neurons in the distance of the AIS from the soma at P16 (10.69 ± 0.64 mm, *n* = 44 cells in HF neurons vs. 6.9 ± 0.31 mm, *n* = 33 cells in LF neurons, *p* < 0.0001, unpaired *t*-test; Figures 3A,C). The refinement of AIS length and location was progressively enhanced through P40 and stabilized in adulthood at P80. Notably, the progressive shortening of the AIS occurred specifically in HF neurons during postnatal development. The AIS of HF neurons was shortened by ∼29.30% by P40 (from P9 to P40, *p* < 0.001, Kruskal–Wallis test with Dunn’s *post hoc* test; Figure 3D). However, there was no significant change in the AIS length of LF neurons, which maintained AIS length throughout adulthood from when the AIS began to be detected at an early postnatal age (from p9 to P80, *p* = 0.2264, Kruskal–Wallis test with Dunn’s *post hoc* test; Figure 3D). The AIS of both HF and LF neurons were relocated more distally from the soma during postnatal development and maintained their spatial distance from the soma in adulthood (HF neurons from P9 to P40, *p* < 0.0001 and LF neurons from P9 to P40, *p* < 0.001, Kruskal–Wallis test with Dunn’s *post hoc* test; Figure 3E). However, the distance of the AIS from the soma was much longer in HF neurons than LF neurons, thus resulting in the tonotopic difference of AIS location in the MNTB. The AIS of HF neurons was located distally, whereas the LF neuron AIS was located closer to the soma. The refinement of AIS length and location progressively developed in postnatal ages. Around P40, AIS length and distance reached a plateau and then stabilized in adulthood, maintaining tonotopic differentiation. The AIS of HF neurons preferentially and dynamically underwent developmental refinement, resulting in the tonotopy of the AIS in the auditory brainstem.

### Sound Modification During Early Postnatal Development Influences AIS Length, Location, and Tonotopic Differentiation

**FIGURE 4:**
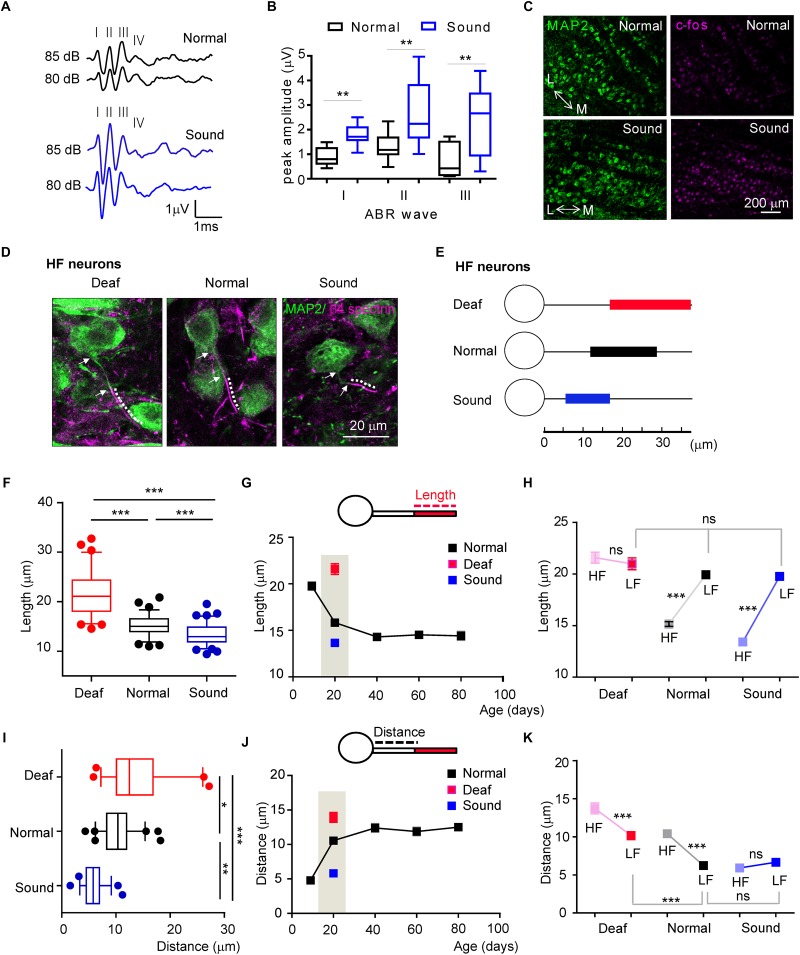
Sound deprivation and stimulation impact developmental refinement of AIS structure, position, and tonotopy in the MNTB. **(A)** Examples of the ABRs of normal (black) and sound stimulated mice (blue) are recorded in response to a click stimulus of sound (80 and 85 dB). Roman numerals indicate peak waves I to IV **(B)** Summary of the amplitude of waves I to III in response to click stimulus (85 dB) in normal and sound stimulated mice at P20. ^∗∗^*p* < 0.01, unpaired *t*-test. **(C)** Immunostaining of the MNTB with c-fos and MAP2 from normal and sound stimulated mouse with the tonotopic axis (M: medial MNTB, L: lateral MNTB). Note, the number of c-fos positive cells is increased in the MNTB of the sound stimulation group. **(D)** HF neurons, immunostained with MAP2 and β4 spectrin from deaf mice (Deaf), age-matched normal hearing mice (Normal), and sound stimulation group (Sound). The dotted line and white arrow indicate AIS length and distance, respectively. **(E)** Diagram of AIS length and location in HF neurons from Deaf (red), Normal (black), and Sound (blue). **(F)** Summary of AIS length in HF neurons from Deaf, Normal, and Sound at P20. ^∗∗∗^*p* < 0.0001, one-way ANOVA with Turkey’s multiple comparison test. **(G)** The plot of AIS length of HF neurons from normal mice from P9 to P80 (black, as shown in Figure 3D), and those from Deaf and Sound at P20. **(H)** Tonotopic differentiation of AIS length between HF and LF neurons in Deaf, Normal, and Sound. ^∗∗∗^*p* < 0.0001, Mann–Whitney *U* test for HF and LF neurons; ns, non-significant, Kruskal–Wallis with Dunn’s *post hoc* test for AIS length of LF neurons from Deaf, Normal, and Sound. **(I)** Summary of AIS location, as defined by the distance from the soma, of HF neurons in Deaf, Normal, and Sound at P20. ^∗∗∗^*p* < 0.001, Kruskal–Wallis with Dunn’s *post hoc* test. **(J)** The plot of AIS location changes during postnatal development in normal mice from P9 to P80 (black, as shown in Figure 3E), and those in Deaf and Sound at P20. **(K)** Tonotopic differentiation of AIS location between HF and LF neurons in Deaf, Normal, and Sound at P20. ^∗∗∗^*p* < 0.0001, either Mann–Whitney *U* test or unpaired *t*-test for HF and LF neurons, Kruskal–Wallis test with Dunn’s *post hoc* test for AIS distance of LF neurons from Deaf, Normal, and Sound.

The structural refinement of the AIS was initiated and progressively developed after hearing onset. To determine whether *in vivo* auditory experience contributes to the initiation and development of structural plasticity and tonotopy of the AIS in the auditory nervous system, we tested the effects of sound deprivation or stimulation on AIS plasticity in the MNTB neurons compared to the age-matched normal hearing mice during postnatal development. As a model of sound deprivation, we utilized a mouse with congenital deafness (hereafter, Deaf) caused by a stereocilia defect due to a mutation of whirlin (DFNB3, Lane, 1963; Xu et al., 2017). For mild sound stimulation, we established a mouse model (hereafter, Sound) exposed to additional sound inputs (80 dB, 16kHz, 3 h./day for 7 days) immediately after hearing onset from P13 to P19, which were chosen to determine whether early sound exposure sped developmental changes in MNTB neurons. We utilized 16 kHz tone sound to target HF neurons and determine if the AIS length during development would be altered by sound experience, because HF neurons displayed a change in AIS length during development. *In vivo* ABRs (*n* = 11 mice in Normal, *n* = 12 mice in Sound) and c-fos staining of the MNTB (*n* = 3 mice for each group) indicated that neuronal activity was significantly increased in the MNTB of the auditory brainstem in the Sound. The amplitude of ABRs was significantly increased in the Sound (wave I: 0.8 ± 0.13 mV in normal mice vs. 1.8 ± 0.13 mV in Sound, *p* = 0.0003; wave II: 1.3 ± 0.15 mV in Normal vs. 2.6 ± 0.35 mV in Sound, *p* = 0.0047; wave III: 0.7 ± 0.24 mV in Normal vs. 2.4 ± 0.42 mV in Sound, *p* = 0.0052, unpaired *t*-test; Figures 4A–C). In the Sound, the number of c-fos + cells was significantly increased (31.7 ± 3.55% in Normal vs. 90.1 ± 3.83% in Sound, *p* = 0.0286, unpaired *t*-test; Figure 4C). Sound deprivation and stimulation significantly altered AIS length and location in HF neurons (Figures 4D,E). Compared to mice in a normal auditory environment (*n* = 6 mice), AIS length was significantly longer as a result of sound deprivation in the Deaf (*n* = 5 mice), whereas AIS length was significantly shorter in the Sound (*n* = 8 mice) compared to the Normal (21.5 ± 0.52 mm, *n* = 67 cells in Deaf vs. 15.1 ± 0.23 mm, *n* = 79 cells in Normal vs. 13.4 ± 0.20 mm, *n* = 94 cells in Sound, *p* < 0.0001, one-way ANOVA with Turkey’s multiple comparison test; Figure 4F). In the developmental time course of AIS length (as shown in Figure 3D), sound deprivation seemed to inhibit the shortening of AIS, while sound stimulation enhanced the shortening of AIS specifically in HF neurons (Figure 4G). However, LF neurons did not exhibit any changes in AIS length in response to either sound deprivation or stimulation (20.9 ± 0.57 mm, *n* = 50 cells in Deaf vs. 19.9 ± 0.37 mm, *n* = 64 cells in Normal vs. 19.7 ± 0.34 mm, *n* = 54 cells in Sound, *p* = 0.3188, Kruskal–Wallis with Dunn’s *post hoc* test; Figure 4H). The AIS structural plasticity of the HF neurons to sound inputs consequently influenced the tonotopic differentiation of AIS length. The analysis of AIS length of HF and LF neurons using animal as the experimental unit indicated the MNTB did not display the tonotopic differentiation of AIS length in Deaf (*n* = 5 mice), whereas in Sound, the tonotopy of AIS length was enhanced (*n* = 8 mice, 2-way ANOVA with Sidak’s multiple comparison test).

In terms of AIS location, the AIS of HF neurons in the Deaf and the Sound were significantly different from the Normal (13.7 ± 0.71 mm, *n* = 54 cells in Deaf vs. 10.4 ± 0.34 mm, *n* = 63 cells in Normal vs. 5.9 ± 0.23 mm, *n* = 57 cells in Sound, *p* < 0.001, Kruskal–Wallis test with Dunn’s *post hoc* test; Figure 4I). The AIS in Deaf mice is more distally located from the soma compared to those in the Normal. However, in the Sound, the location of the AIS was more proximal to the soma. This proximal shift of the AIS position resulted in an increase in the spatial distance between the proximal edge of AIS and the soma in the Deaf, whereas there was a significant decrease in this distance in the Sound compared to the normal age-matched mice (as shown in Figure 3E) in the natural auditory environment (Figure 4J). AIS distance was increased in LF neurons of the Deaf, but no significant change was observed in the Sound (6.2 ± 0.24 mm, *n* = 74 in Normal vs. either 10.1 ± 0.51 mm, *n* = 43 cells in Deaf, *p* < 0.001, or 6.6 ± 0.38 mm, *n* = 67 cells in Sound, p > 0.05, Kruskal–Wallis test with Dunn’s *post hoc* test; Figure 4K). In the analysis of AIS location of HF and LF neurons using animal as the experimental unit, there was no tonotopic differentiation of AIS location in the Sound whereas in Deaf the tonotopy of AIS location existed (*n* = 8 mice vs. *n* = 6 mice for Normal, and *n* = 5 mice for Deaf groups, 2-way ANOVA with Sidak’s multiple comparison test). These results indicate that excessively altered sound inputs in different auditory environments critically impact AIS structure, location, and tonotopy in the mouse auditory brainstem during development.

### After the Stabilization of AIS Structure in Adulthood, Alteration of Sound Input Disrupts the Structural and Spatial Stability of the AIS Tonotopy

**FIGURE 5:**
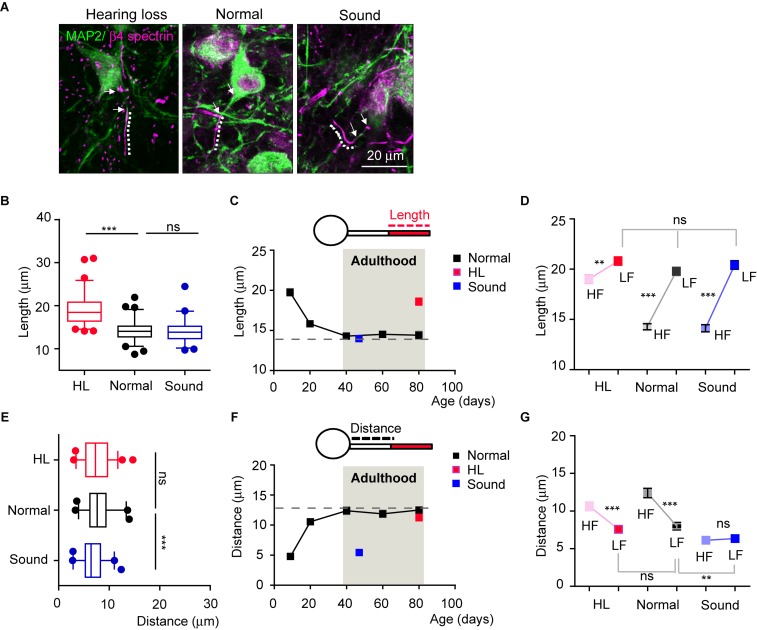
Auditory experience regulates AIS structural and spatial plasticity in adulthood. **(A)** HF MNTB neurons, immunostained with MAP2 and β4 spectrin from hearing loss (HL), Normal, and Sound. The dotted line and white arrow indicate AIS length and distance, respectively. **(B)** Summary of AIS lengths in HF neurons from HL, Normal, and Sound. ^∗∗∗^*p* < 0.001, Kruskal–Wallis test with Dunn’s *post hoc* test. **(C)** The plot of AIS length in HF neurons from normal mice from P9 to P80 (black, shown in Figure 3D), and those from HL (at P80, red), and Sound (at P45, blue). **(D)** Tonotopy of AIS length between HF and LF neurons from HL, Normal, and Sound groups. ^∗∗^*p* < 0.01, ^∗∗∗^*p* < 0.0001, Mann–Whitney *U* test for HF and LF neurons; ns, non-significant, Kruskal–Wallis with Dunn’s *post hoc* test for AIS length of LF neurons from HL, Normal, Sound. **(E)** Summary of AIS distance in HF neurons from HL (red), Normal (black), and Sound (blue). ^∗∗∗^*p* < 0.0001, one-way ANOVA with Turkey’s multiple comparison test. **(F)** The plot of AIS distance in HF neurons from Normal from P9 to P80 (black, shown in Figure 3E), and those from HL (red) and Sound (blue). **(G)** Tonotopy of AIS location between HF and LF neurons from HL, Normal, and Sound. ^∗∗∗^*p* < 0.0001, unpaired *t*-test for HF and LF neurons; ^∗∗^*p* < 0.01, one-way ANOVA with Turkey’s multiple comparison test for AIS distance of LF neurons from hearing loss, normal, sound group.

We next questioned whether auditory neurons maintained structural plasticity of the AIS following changes in the auditory environment in adulthood, even after AIS length, location, and tonotopy are stabilized. We tested adult mice (4 mice per each group) that experienced sudden hearing loss after blast exposure (Choi et al., 2015, see in method section) and those that experienced additional mild sound stimulation for a week (16kHz, 3 h/day, for 7 days). The AIS of HF neurons was significantly elongated in adult mice that experienced hearing loss (at P80), comparing to age-matched normal mice. Interestingly, there was no alteration in AIS length in the Sound (14.2 ± 0.30 mm, *n* = 66 cells in the Normal vs. either 19.0 ± 3.53 mm, *n* = 64 cells in hearing loss, *p* < 0.001 or 14.1 ± 0.34 mm, *n* = 57 cells in Sound, *p* > 0.05, Kruskal–Wallis test with Dunn’s *post hoc* test; Figures 5A,B). In the developmental plot of AIS length (as shown in Figure 3D), the length at around P40 reached a steady state of ∼15 mm, which could be the maximum shortening of AIS during development (Figure 5C). Thus, further mild sound stimulation cannot enhance the shortening of the AIS beyond this plateau of AIS length after development. Consistent with the results seen in postnatal development, AIS length in LF neurons was not affected by either hearing loss or the Sound in adulthood (20.8 ± 0.41 mm, *n* = 55 cells in hearing loss vs. 19.7 ± 0.43 mm, *n* = 57 cells in Normal vs. 20.4 ± 0.42 mm, *n* = 56 cells in Sound; *p* = 0.2092, Kruskal–Wallis test with Dunn’s *post hoc* test; Figure 5D). Thus, elongated AIS specifically in HF neurons in the hearing loss model resulted in the loss of tonotopic segregation of AIS length in the MNTB (*n* = 4 mice for each group, 2-way ANOVA with Sidak’s multiple comparison test; Figure 5D).

AIS location of HF neurons in the hearing loss model was not changed, whereas the AIS was relocated close to the soma in the Sound, compared to the Normal (12.3 ± 0.62 mm, *n* = 40 cells in the Normal vs. either 10.6 ± 0.57 mm, *n* = 53 cells in hearing loss, *p* > 0.05 or 6.1 ± 0.32 mm, *n* = 37 cells in the Sound, *p* < 0.0001, one-way ANOVA with Turkey’s multiple comparison test; Figure 5E). It is also interpreted that adult HF neurons have reached a maximum distance between the AIS and the soma (∼13 mm; Figure 5F). This plateau of the developmental plot of the spatial distance between the AIS and soma might be the limit for the physical distance to generate APs by the integration of somatic and dendritic signals (Hamada et al., 2016). AIS location of LH neurons was dynamically altered in the Sound and affected the tonotopy of AIS location (2-way ANOVA with Sidak’s multiple comparison test; Figure 5G). The results demonstrated that, after developmental refinement, AIS plasticity in adulthood following auditory experience occurred only within the structural and spatial boundaries of the AIS.

### AIS Structural Alterations Are Distinctively Observed in Age-Related Hearing Loss

**FIGURE 6:**
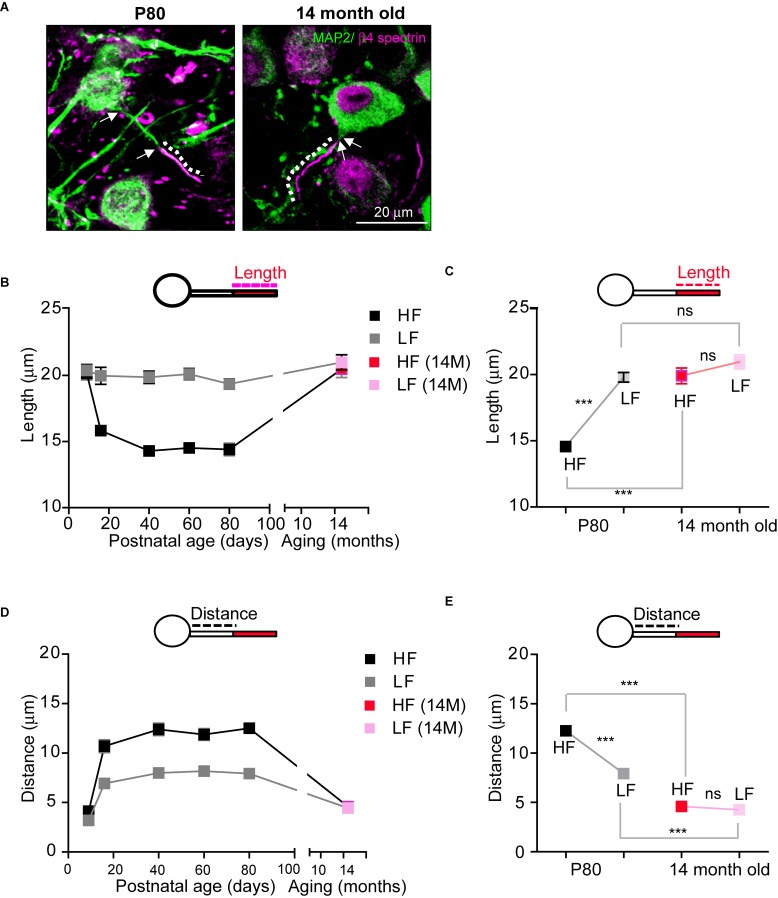
The structural stability of the AIS of MNTB neurons is disrupted in aged mice. **(A)** HF neurons were immunostained with MAP2 and β4 spectrin in adulthood (P80) and in an aged mouse (14-month-old; 14M). The dotted line and white arrow indicate AIS length and distance, respectively. **(B)** The plot of AIS length in HF (black) and LF (gray) neurons through lifetime from P9 to 14M and AIS length of HF (red) and LF (pink) in aged mice. **(C)** Tonotopy of AIS length in MNTB neurons from adult (at P80, black) and aged (14M, magenta) mice. ^∗∗∗^*p* < 0.0001, Mann–Whitney *U* test. **(D)** The plot of AIS location in HF (black) and LF (gray) neurons through lifetime from P9 to 14M and AIS location of HF (red) and LF (pink) in aged mice. **(E)** Tonotopy of AIS location from adult (at P80, black) and aged (14M, red) mice. ^∗∗∗^*p* < 0.0001, unpaired *t*-test.

Age-related hearing loss critically changes auditory brain structure in humans (Lin et al., 2014; Peelle et al., 2011). In the elderly, a decline in hearing sensitivity is one prominent phenomenon of aging. Thus, we examined how age-related hearing loss impacts AIS structure in the auditory nervous system using aged mice (14-month-old; 14M, C57BL/6 mice), which showed an elevated threshold and reduced amplitude of ABRs (Hunter and Willott, 1987). In aged mice (*n* = 4 mice), AIS length in HF neurons was significantly increased, compared to those in normal adult mice (14.5 ± 0.29 mm, *n* = 96 cells in adult mice at P80, as shown Figure 3 vs. 19.9 ± 0.59 mm, *n* = 60 cells in aged mice (14M), *p* < 0.0001, Mann–Whitney *U-*test; Figures 6A,B). However, LF neurons in aged mice did not show any difference in AIS length compared to normal adult mice (19.7 ± 0.36 mm, *n* = 84 cells in adult mice vs. 20.9 ± 0.53 mm, *n* = 57 cells in aged mice, *p* = 0.0745, Mann–Whitney *U-*test; Figure 6B). Although there was a significant difference in AIS length between HF and LF neurons, this elongated AIS of HF neurons lessened the tonotopic segregation of AIS length in aged mice (HF vs. LF neurons of either adult mice, *p* < 0.0001 or aged mice, *p* = 0.0669, Mann–Whitney *U-*test; Figure 6C).

As for AIS location, the AIS of both HF and LF neurons in aged mice were located more proximally to the soma, decreasing the spatial distance between the AIS and soma (HF: 12.2 ± 0.42 mm, *n* = 78 cells in adult mice vs. 4.5 ± 0.25 mm, *n* = 50 cells in aged mice, *p* < 0.0001, Mann–Whitney *U* test and LF: 7.9 ± 0.33 mm, *n* = 66 cells in adult mice vs. 4.2 ± 0.23 mm, *n* = 59 cells in aged mice, *p* < 0.0001, unpaired *t*-test; Figure 6D). However, the change in AIS distance from the soma was greater in HF neurons than LF neurons, and thus the tonotopy of AIS location decreased in aged mice (HF vs. LF neurons of either adult mice, *p* < 0.0001, Mann–Whitney *U* test or aged mice, *p* = 0.3440, unpaired *t*-test; Figure 6E). Taken together, these data demonstrate that AIS structure was elongated and relocated close to the soma, and the tonotopy of AIS structure and location was significantly impaired in aged mice with hearing loss.

### Sound Modification Influences Firing Pattern and Excitability of MNTB Neurons

**FIGURE 7:**
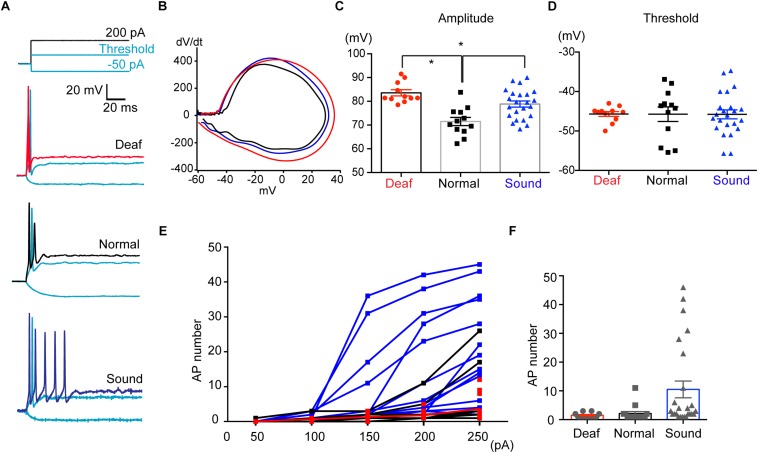
Sound stimulation increases the excitability of HF neurons in the MNTB. **(A)** Representative traces of APs evoked by step-like current injections (-50 pA, threshold current, 200 pA) from Deaf, Normal, and Sound groups (P17- P20). **(B)** Representative plot of dV/dt of AP against membrane potential in Deaf (red), Normal, and Sound. **(C,D)** Summary of the amplitude and threshold of APs, evoked by 200 PA current injection, from Deaf, Normal, and Sound groups. ^∗^p < 0.05, one-way ANOVA with Turkey’s multiple comparison test or Kruskal–Wallis test with Dunn’s *post hoc* test. **(E)** The plot of spike number evoked by current injections (50 pA to 250 pA, 100 ms) in HF neurons from Deaf, Normal, and Sound groups. **(F)** Summary of spike number evoked current injection (200 pA). ^∗^*p* < 0.05, Kruskal–Wallis with Dunn’s *post hoc* test.

AIS structure in HF neurons underwent dynamic refinement during development and aging, as well as in response to various auditory experiences. Next, we explored how sound modification influence physiological properties of HF neurons of MNTB as well as the structural alterations of the AIS. Using whole-cell patch clamp recordings, we recorded APs in MNTB neurons from mice that experienced different sound inputs (P17-P20, Deaf, Normal, or Sound, Figure 7A). In the current clamp recordings, there was no difference in the baseline potential, which was around −70 mV with ∼−20 pA injection (−72.1 ± 0.83 mV for Normal, *n* = 17 cells, −72.1 ± 0.77 mV for Sound, *n* = 18 cells, −70.7 ± 0.71 for Deaf, *n* = 15 cells). We examined the waveform of single APs and found that the AP amplitude was increased in both Sound and Deaf compared to the Normal (*n* = 12 cells in Normal vs. either *n* = 11 cells in Deaf, *p* = 0.174 or *n* = 23 cells in the Sound, *p* = 0.0215, one-way ANOVA with Dunnett’s multiple comparison test; Figures 7B,C and Supplementary Data-Table 1). Other parameters of AP waveforms, including half-width, AP threshold, rheobase current, input resistance, membrane constant were not significantly different between the three groups (Figure 7D and Supplementary Data-Table 1). In addition, we analyzed the number of APs in response to a 100 ms current injection of 200 pA in HF neurons in the Deaf, the Normal, and the Sound. The number of APs was significantly increased in the Sound (1.5 ± 0.29, *n* = 14 cells in Normal vs. 10.5 ± 2.91, *n* = 25 cells in Sound, *p* < 0.01, Kruskal–Wallis test with Dunn’s *post hoc* test; Figures 7E,F). Incremental current injection did not increase AP number in the HF neurons of the Deaf, which was similar to the response in those of the Normal (2.1 ± 0.68, *n* = 15 cells in Normal vs. 1.5 ± 0.23, *n* = 12 cells in Deaf, *p* > 0.05, Kruskal–Wallis test with Dunn’s *post hoc* test; Figures 7E,F). Although AP amplitude was increased in both Sound and Deaf mice, only sound stimulation increased neuronal excitability of HF neurons in the MNTB.

### Computer Simulation Indicates That Structural Changes of AIS Directly Influence the Firing Properties of MNTB Neurons

**FIGURE 8:**
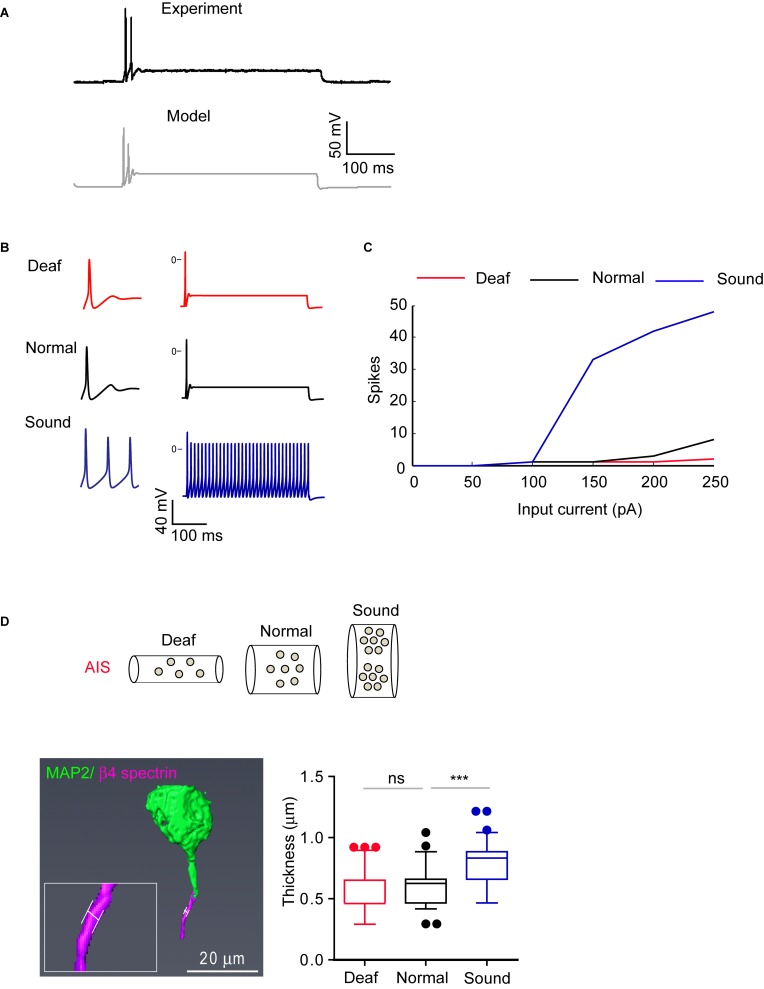
AIS structural changes in sound stimulated group are associated with alteration in firing properties. **(A)** Representative AP evoked by current injection (200 pA) from MNTB neurons in normal hearing mouse (black, experiment) and modeling of MNTB neuron from a computer simulation (gray, model). **(B)** Representative traces of the model when stimulated by 200 pA of constant current with parameters that correspond to the Deaf, Normal, and Sound. **(C)** Summary of AP number evoked by current injection (50 pA–250 pA) from Deaf (red), Normal (black), and Sound (blue) using computer simulation (top). **(D)** Expected AIS structure with ion channel expression from Deaf, Normal, and Sound (bottom). Note, axon diameter is changed in sound modified groups. Bottom, an example of 3D reconstruction of MNTB neuron at P20 mice immunostained with β4 spectrin and MAP2. The white line indicates the thickness of AIS (left). Summary of AIS diameter from Deaf (red), Normal (black), and Sound (blue). ^∗∗∗^*p* < 0.0001, one-way ANOVA with Turkey’s multiple comparison test.

To test the effects of AIS structural changes on the excitability of MNTB neurons of Deaf, Normal, and Sound, we used a computer model. We established a model of an MNTB neuron to replicate the spiking response to somatic current injection in Normal (Figure 8A and Supplementary Data-Table 2). In order to replicate the changes in excitability, we had to make two assumptions. The first is that the density of the channels in the AIS is inversely proportional to its length, which is altered in the different auditory experience conditions compared to control.

(1) $$g_{f}=\frac{L_{N}}{L_{f}}\,g_{N}$$

Where L_*N*_ is the length of the AIS of Normal; L_*f*_ is the final length of the AIS of Deaf or Sound; g_*N*_ is the channel density (S/cm2) of Normal; and g_*f*_ is the channel density of Deaf or Sound. Secondly, we assume that the total surface of the AIS from Sound or Deaf is identical to the surface area of the Normal:

(2) $$d_{f}=\frac{L_{N}}{L_{f}}\,d_{N}$$

Where d_N_ is the diameter of the AIS from Normal; and d_f_ the AIS diameter of Sound or Deaf. Finally, we obtained the equation (3) by combining the equations 1 and 2:

(3) $$g_{t\,o\,t\,a\,l_{f}}=\pi \,\frac{L_{N}}{L_{f}}\,d_{N}\,L_{f}\,\frac{L_{N}}{L_{f}}\,g_{N}={\left(\frac{L_{N}}{L_{f}}\right)}^{2}\,g_{t\,o\,t\,a\,l_{N}}$$

Where g_totalN_ and g_totalf_ are the total conductance (in Siemens) of the Normal and Sound, respectively. We used equation (3) to calculate the total conductance for each channel of the AIS from the Deaf and Sound. For each case, we calculated the excitability response to somatic current injections. The models replicate our findings that HF neurons from the Sound had shorter AIS length and distance and increased excitability (Figure 8B). Assuming only morphological changes, a fixed morphology with changes in the density of ion channels, or one of the above processes at a time failed to reproduce the data (Supplementary Data-Table 2). Thus, changes in AIS morphology can affect as well as be influenced by a change in the excitability of MNTB neurons including AP amplitude in different auditory environments.

Furthermore, the simulation reveals axon diameter as an additional key condition for structural changes to reproduce firing patterns from different models (Deaf, Normal, and Sound). The model predicted a 23% increase in the diameter of the axon in the Sound compared to the Normal mice (Figures 8B,C). To verify this finding, we assessed the diameter of AIS in MNTB neurons (P20 mice) that are immunostained with β4 spectrin and MAP2 using 2D Z-stack compression and 3D reconstruction of confocal images (Figure 8D). The AIS diameter from Sound was significantly increased by 31% compared to the Normal (0.60 ± 0.018 mm, *n* = 69 cells from 3 Normal mice vs. either 0.55 ± 0.020 mm, *n* = 66 cells from 3 Deaf mice, *p* > 0.05 or 0.79 ± 0.018 mm, *n* = 90 cells from 3 Sound, *p* < 0.0001, one-way ANOVA with Turkey’s multiple comparison test; Figure 8D). Sound stimulation alters AIS length, location, and diameter, resulting in an increase in excitability.

## Discussion

### Heterogeneity of AIS Structure Across Various Brain Regions in Different Species

Recent studies have demonstrated AIS plasticity during development (Gutzmann et al., 2014; Kuba et al., 2014), neuronal activity alterations (Grubb and Burrone, 2010; Wefelmeyer et al., 2015), sensory deprivation (Kuba et al., 2010; Gutzmann et al., 2014), and brain disorders (Hsu et al., 2014). Furthermore, heterogeneity of the AIS has been considered in various cell types, brain regions, and across different species (Wefelmeyer et al., 2015; Höfflin et al., 2017). In the auditory system, development and plasticity of AIS structure have been studied in the nucleus laminaris (NL) and nucleus magnocellularis (NM) of the chick brainstem (Kuba et al., 2006, 2010, 2014). Consistent with what has been shown in chick NL neurons, mouse MNTB neurons display a similar tonotopic differentiation of AIS structure and AP waveform (Figure 2). HF neurons, which had a shorter AIS length and longer distance from the AIS to the soma, had smaller amplitude APs than those in LF neurons. However, the chick AIS from both LF and HF neurons was shortened during development (Kuba et al., 2014), whereas, in the mouse, MNTB specifically HF neurons significantly underwent AIS refinements until early adulthood. The developmental refinement of the AIS in HF neurons affects spike waveform in mouse MNTB neurons as well as in the chick (Kuba et al., 2014). Discrepancies in AIS plasticity data between the chick and mouse might be related to differences in audiograms from chick (from 2 Hz to 9 kHz, Hill et al., 2014) and mouse (from 4 kHz to 64 kHz, Greich and Strutz, 2012). The MNTB is a well-developed nucleus in rats and mice, which hear higher frequency sounds than chicks. The chick NL is more comparable to the medial superior olivary (MSO) in rodents that preferably hear lower frequency sound, such as gerbils (Greich and Strutz, 2012).

Sound-evoked activity is important for the developmental refinement of the AIS. In congenital deafness, HF neurons of the mouse MNTB had a longer AIS compared to those in age-matched normal mice. Sound input is critical for developmental shortening of the AIS, and thus sound deprivation in deafness inhibited this shortening and left longer immature AIS. AIS plasticity was more dynamic in HF neurons. In the mouse MNTB, AIS length of LF neurons was very consistent throughout life, regardless of sound modifications either during development or in adulthood, but AIS location of LH neurons was dynamically moved in response to sound modifications in the mouse MNTB. The AIS of LH neurons moved proximal to the soma in sound stimulation but moved distally in sound deprivation. In cultured hippocampal neurons and pyramidal cells, the AIS was moved away from the soma and relocated distally during prolonged depolarization (Grubb and Burrone, 2010; Wefelmeyer et al., 2015). In these cells, the dynamic shift of the AIS position contributes to the modulation of neuronal excitability (Grubb and Burrone, 2010). A possible explanation for the proximal movement of the AIS of MNTB neurons in sound stimulation could be cell-type specificity of GABAergic neurons. Contradictory to non-GABAergic neurons, GABAergic olfactory bulb interneurons show proximal lengthening of the AIS, which relocated closer to the soma after chronic 24h depolarization (Chand et al., 2015). Therefore, cell-type or brain-region specific structural plasticity of the AIS could explain these different responses from different brain areas.

### The Structural Plasticity of AIS Throughout Life From Development to Aging

**FIGURE 9:**
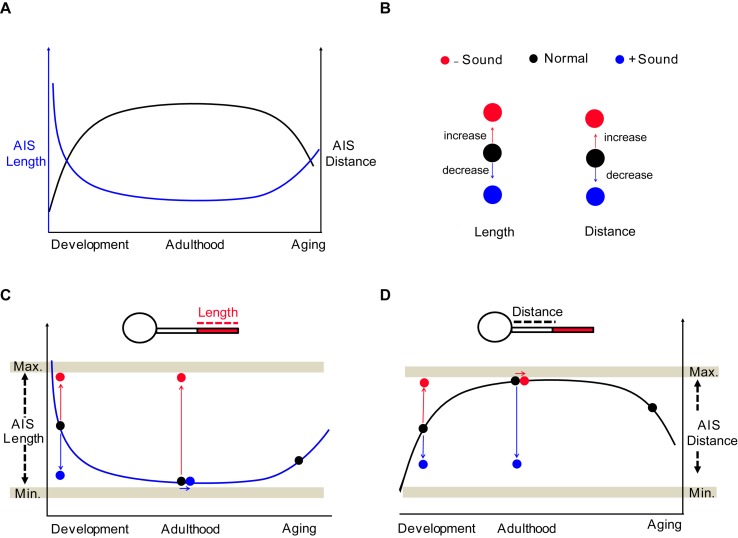
Summary of the structural and spatial plasticity of the AIS in the auditory brainstem throughout life. **(A)** Diagram of AIS structural remodeling throughout the lifespan from neonatal development to aging. The plot of AIS distance (black) and length (blue) show a parabola and inverse parabola. **(B)** Effects of sound inputs on the structural plasticity of AIS; deaf mice or HL model (−Sound, red) and sound stimulation (+ Sound, blue). **(C,D)** The impact of sound experiences on AIS structures throughout the lifespan. The structural plasticity occurs within the hypothetical minimum and maximum boundaries, which are indicated by Min. and Max. (gray horizontal bar).

Structural and functional properties of the mammalian brain change across the lifespan (Freitas et al., 2013; Yeatman et al., 2014). Understanding the profile of AIS structure in the perspective of an entire lifetime can provide essential information to identify the intrinsic molecules or signaling pathways underlying the regulation of AIS structure at different ages. The AIS undergoes shortening during development in the chick auditory brainstem and in monkey prefrontal cortex (Cruz et al., 2009; Kuba et al., 2014). In the visual cortex of mice *in vivo*, AIS length steadily increases until P15, and then shortens dramatically after eye-opening until the beginning of the critical period of cortical ocular dominance plasticity at P21 (Gutzmann et al., 2014). Developmental plasticity of AIS length and position may be associated with the individual functional state of a given neuron in specific brain regions (Gutzmann et al., 2014; Schlüter et al., 2017). In the mouse auditory brainstem, we found that the plot of AIS location (or length) formed a parabola (or inverse parabola) when plotted against age from neonatal development to aging (Figure 9A). In postnatal development, AIS dynamically underwent shortening and then stabilization of AIS structure and location, resulting in a steady plateau of the developmental plot of AIS length and location in adulthood (Figure 9A). The structural plasticity of the AIS occurs within a specific range, with a minimum and maximum in terms of AIS length and location, indicating that the AIS is structurally resilient (Figures 9B–D). In aging, if the structural resilience of the AIS is lost, similar to the loss in elasticity of a spring over time, the AIS might be altered, as we observed in the auditory brainstem (Figure 6). The mechanism of this elongation in aging is different from AIS elongation due to homeostatic processes to compensate for decreased neuronal activity (Gründemann and Häusser, 2010; Wefelmeyer et al., 2016). The levels of ankyrinG, spectrin, and actin in the AIS are reduced in aged mice (Bahr et al., 1994). Primary visual cortical neurons in aged rats displayed shortening of the AIS and reduction of Na_V_1.6 channel expression along the AIS, which accompanies enhanced neuronal activity (Ding et al., 2018). A decline in sensory inputs seems to impair the structural stability of the AIS in the sensory system. However, the implication of age-related alterations in the AIS or the link between morphological changes in the AIS and neuronal excitability remains unclear in the aged brain. Further investigation of molecular mechanisms targeting AIS re-organization during aging will provide a new strategy for central auditory disorders following age-related hearing loss.

### AIS Changes in Auditory Environmental Modification

In modern society, there is increased exposure to sensory inputs in technologically advanced environments. Increase in chronic exposure to various sounds (from mild to severe) might affect the development and maturation of the auditory nervous system. Sound stimulation facilitated AIS shortening during development, but did not further change AIS length in adulthood. The structural plasticity of AIS driven by sound inputs might stay within a particular range, preventing the AIS from becoming overly shortened (Figures 9B–D). Regarding the AIS position, either during development or in adulthood, sound stimulation proximally moved the AIS of MNTB neurons. This spatial relocation was contrary to previous studies in cultured hippocampal neurons or organotypic brain slices, showing a distal shift of the AIS along the axon in response to elevated neuronal activity (Grubb and Burrone, 2010; Wefelmeyer et al., 2015), indicating a cell-specific effect of sound stimulation in the auditory system. These results demonstrate significant alterations in cellular structures of the auditory brain as a result of sound stimulation.

In the mammalian auditory system, the superior olivary complex is the first major convergence point for binaural information, where the MNTB-the lateral superior olive (LSO) circuit contributes to binaural intensity processing and lateralization (Heffner and Masterton, 1990). Computationally, changes in excitability could affect the non-monotonic responses to regular spiking input in MNTB cells (Arnoldt et al., 2015). Increased ABRs and c-fos signal in the MNTB shown in sound stimulation mice suggest that an increase in spontaneous activity and/or changes in the peripheral plasticity upstream of the auditory brainstem can occur in sound enhanced condition. In deaf mice, it is unclear how sound deprivation affect spontaneous activity in the auditory brainstem. Our model might be of interest to interaural sound processing where models of MNTB neurons are needed (Ashida et al., 2017). Thus, severe alterations in AIS structure in the MNTB might impact sound localization.

### The Physiological Role of AIS Structural Refinement in Intrinsic Excitability of Auditory Neurons

A key question is how AIS plasticity drives the adaptation of neuronal excitability. The AP amplitude of MNTB neurons is critical for their fast spiking properties (Kim and von Gersdorff, 2012). It is hypothesized that the AP amplitude is mediated by ion channel properties and location. This is consistent with findings from Leão et al. (2005) showing that the location of sodium channels in the nerve terminal impacts the AP waveform. The computer model predicts that the location of Na channels closer to the terminal may result in a larger AP with higher amplitude (Leão et al., 2005). Here our model suggests that AIS length and location can affect AP amplitude, which is associated with fast spiking properties of MNTB neurons in the auditory brainstem. Unlike our expectation in the model, our whole-cell recordings showed a larger AP with higher amplitude in MNTB neurons from both deaf and sound stimulation mice. One possible explanation is that several factors including non-AIS-related features such as soma size (Weatherstone et al., 2017) or somatic Na^+^ or K^+^ channel density (Leao et al., 2006; Leão et al., 2008) are associated with these alterations in MNTB excitability in deaf and sound stimulation mice. Another possibility is that congenitally the cochlea dysfunction in deaf mice can influence central ion channel expression during development (Leao et al., 2006). The physiological role of non-AIS related features and how they act synergistically with AIS plasticity to mediate intrinsic excitability of auditory neurons needs to be addressed in future studies. In the sound stimulation group, MNTB neurons had a shorter AIS relocated closer to the soma and displayed an increase in spike number in response to current injection compared to the normal and deaf mice. The greatest level of excitability possible in a neuron occurs when the AIS is at a certain distance from the soma, but there is a maximum distance necessary to overcome the charge dissipation and generate APs (Hu and Jonas, 2014; Hamada et al., 2016). In the sound stimulation group, the proximal location of the AIS with a shorter length may maximize the excitability of MNTB neurons, isolating the AIS from somatodendritic compartments and reducing the shunting conductance (Gulledge and Bravo, 2016; Yamada and Kuba, 2016). Differential responses of the AIS to neuronal activity may be affected by stimulation conditions (e.g., *in vitro* stimulation or *in vivo* environmental stimulation) and by the duration of the stimuli (e.g., short-term or long-term). This could explain the discrepancies between our study and previous studies showing a distal shift along the axon in elevated neuronal activity (Grubb and Burrone, 2010; Wefelmeyer et al., 2015). Understanding the physiological consequences or homeostatic adaptation processes in response to sound deprivation or stimulation in the central auditory system throughout life is critically important for developing targeted strategies to remedy potential long-term deficits of the auditory brain at specific ages.

## Data Availability Statement

All datasets generated for this study are included in the manuscript/Supplementary Files.

## Ethics Statement

The animal study was reviewed and approved by the University of Texas Health Science Center, San Antonio (UTHSCSA) Institutional Animal Care and Use Committee protocols.

## Author Contributions

EK performed the experiments, analyzed the data, and wrote the manuscript. CF performed the computer modeling. FS performed the computer modeling, discussed the data, and wrote the manuscript. JK conceived and performed the experiments, analyzed the data, wrote the manuscript, provided the financial support, and supervised the study.

## Conflict of Interest

The authors declare that the research was conducted in the absence of any commercial or financial relationships that could be construed as a potential conflict of interest.
