# Supplementary material for: Impact of Auditory Experience on the Structural Plasticity of the AIS in the Mouse Brainstem Throughout the Lifespan
Source: Front Cell Neurosci. 2019 Oct 15;13:456. doi: 10.3389/fncel.2019.00456 (PMC6813928; doi:10.3389/fncel.2019.00456)
Supplement: Supplementary file 1 [file Data_Sheet_1.docx]

**Supplementary Materials**

**Impact of auditory experience on structural plasticity of the AIS in the mammalian brain throughout the lifespan**

Eun Jung Kim^1^, Chenling Feng^2^, Fidel Santamaria^2^, and Jun Hee Kim^1*^

^1^The Department of Cellular and Integrative Physiology, UT Health San Antonio, Texas 78229, USA, ^2^The Department of Biology, University of Texas San Antonio, Texas 78249, USA

*Corresponding author: Jun Hee Kim, Ph.D. (kimjh@uthscsa.edu)

**Supplementary Table 1.** Analysis of single action potential (AP)

|  |  | **Deaf** | **Normal** | **Sound** |
| --- | --- | --- | --- | --- |
| **AP at 200pA** | **Amplitude (mV)** | 83.58 ± 1.306 (N=11) | 71.52 ± 1.730  (N=12) | 78.87 ± 1.291  (N=23) |
|  | **Threshold (mV)** | -45.68 ±0.606 | -45.74 ± 1.853 | -45.77 ± 1.136 |
|  | **Half Width (ms)** | 0.4 ± 0.03 | 0.5 ± 0.04 | 0.4 ± 0.02 |
|  | **Rise time (μs)** | 176.3 ± 7.60 | 201.2 ± 11.92 | 185.1 ± 9.61 |
|  | **Input Resistance** | 0.3 ± 0.04 | 0.2 ± 0.02 | 0.2 ± 0.01 |
|  | **Membrane-constant (Tau, ms)** | 5.5 ± 0.51 | 5.7 ± 0.39 | 6.6 ± 0.46 |

**Supplementary Table 2**

We implemented a multi-compartmental model of an MNTB neuron based on a previous publication([1](#_ENREF_1), [2](#_ENREF_2)). The model consisted of a soma, a primary dendrite, an axonal hillock, and an axonal initial segment (AIS). The reference model was built to replicate the structure and physiology of HF MNTB neurons in the normal sound environment. The lengths and diameters of each segment are described in Table 1.

Table 2-1. MNTB neuron compartmental model under normal sound environment.

| **Segment** | **Length (µm)** | **Diameter (µm)** |
| --- | --- | --- |
| Dendrite | 40 | 3 |
| Soma (spherical) |  | 20 |
| Hillock | 10.4 | 2 |
| AIS | 13 | 2 |

The MNTB model had the following conductances: leak, sodium (Nav1.1), potassium (KV1.1 and KV3.1), and HCN. The kinetics were obtained from a previous publication. The conductance density were adjusted to replicate the action potential shape and excitability experimentally measured for animals raised in the normal sound environment. For this, we used a Monte Carlo approach that generated random vectors over the parameter space of all the conductances. All simulations consisted in delivering current steps to the soma from 0 to 0.3 nA. For each simulation, we calculated the number of spikes, the height, the width at half-height, and the threshold of the first spike (Figure 1). Table 2 shows the density of each channel and Table 3 shows the kinetics of each conductance.

Table 2-2. Maximum conductance for each channel expressed in the MNTB neuron model. All in S/cm^2^x10^-3^.

| Conductance | dendrite | Soma | hillock | AIS |
| --- | --- | --- | --- | --- |
| Leak | 0.08 | 0.08 | 0.08 | 0.08 |
| NaV1.1 |  |  |  | 362.53 |
| KV3.1 |  | 186.42 |  |  |
| KV1.1 |  |  |  | 2.95 |
| H channel | 0.04 | 0.06 |  |  |

Table 2-3. Kinetics of membrane conductances in the MNTB neuron model. V is the membrane potential. All kinetics are Hodgkin-Huxley.

| Name | State | n | Model |
| --- | --- | --- | --- |
| NaV1.1 | Activation | 3 | $\alpha_{m}=76.4e^{0,037v}$ $\alpha_{h}=0.00013e^{-0.1216v}$  $\beta_{m}=6.930852e^{-0.043v}$ $\beta_{h}=1.999e^{0.0384v}$ |
|  | Inactivation | 1 |  |
| Kv1.1 | Activation | 1 | $\alpha_{l}=1.2e^{0.03512v}$ $\alpha_{r}=0.0438e^{-0.0053v}$  $\beta_{l}=0.2248e^{-0.0319v}$ $\beta_{r}=0.0562e^{-0.0047v}$ |
|  | Inactivation | 1 |  |
| Kv3.1 | Activation | 1 | $\alpha_{n}=0.2719e^{0.04v}$ $\alpha_{p}=0.0073e^{-0.1942v}$  $\beta_{n}=0.1974e^{0v}$ $\beta_{p}=0.0936e^{-0.0058v}$  $i_{k}= \bar{g}n^{3}(1-\gamma+\gamma p)(v-e_{k})$  $\gamma=0.1$ |
|  | Inactivation | 1 |  |
| H channel | Activation | 1 | $\alpha=\frac{0.63}{1000}e^{-0.063\left( v+73.1 \right)}$  $\beta=\frac{0.63}{1000}e^{0.078\left( v+73.2 \right)}$ |

The model was implemented in Neuron-Python and analyzed in custom routine in Matlab (Natick, MA). All simulation scripts, results, and analyses are deposited in ModelDB (<https://senselab.med.yale.edu/ModelDB/default.cshtml>, accession number: 259482) and GitHub (github.com/SantamariaLab).

The model was built to replicate the spike amplitude, the spike width at half-amplitude, and the threshold (Figure S1 compare to Figure 2). We calculated the spike threshold by using a phase-plane method where we plotted the speed of the voltage against the voltage. We found the membrane voltage that corresponded to a given speed (60 mV/ms)([3](#_ENREF_3)). We also replicated the response to current steps that characterized the intrinsic excitability response (see below and Figure 7). Once the model reproduced the spiking response of the MNTB neuron under the normal sound environment (Figure S1 compare to Figure 2), we determined how the structural and physiological properties of the cell change in the noise and deaf conditions. First, we started by varying the length of the AIS and hillock, independently. We covered the range of AIS lengths, from 20 µm in the deaf conditions, to 10 µm in the sound. We kept all other parameters as in the normal sound environment condition (Figure S2A). This shows that the excitability of the cell, measured as the number of spikes over a 100 ms period for a range of current steps, increases as the length increases. This is the opposite behavior than in the experiments. Changing the length of the hillock (from 4 µm to 14 µm) had a minimum effect in the excitability response of the cell (Figure S2B). This is because the hillock only has passive properties.

The experiments showed that the excitability of the MNTB cell is inversely related to the length of the AIS. This could be because the cell maintains the same total conductance of the sodium and potassium channels as in the normal condition. If $g_{n}$is the conductance density, of sodium or potassium channels, in the AIS in the normal condition, then the final density in the deaf or sound conditions ($g_{f}$) follows this relationship:

${g_{f}\pi d_{n}l_{f}=g}_{n}\pi d_{n}l_{n}$(1)

Where $d_{n}$ is the diameter in the normal condition, which remains fixed, and $l_{n}$ and $l_{f}$ are the normal (16 µm) and final lengths, respectively. This results in

$g_{f}=g_{n}\frac{l_{n}}{l_{f}}$ (2)

We ran simulations with different AIS lengths and scaled the sodium and potassium channel conductances accordingly to Eq. 2 (Figure S2C). This analysis shows that the excitability of the cell was practically unchanged. We obtained a similar result when keeping the total area of the AIS equal to the area of the AIS in the normal condition (Figure S3C):

$d_{f}=d_{n}\frac{l_{n}}{l_{f}}$ (3)

Where $d_{n}$ and $d_{f}$ are the normal and final diameters, respectively. This is not surprising since the total conductance of the AIS in the both cases is

$G_{AIS,f}=g_{f}\pi d_{f}l_{f}$ (4)

Where $G_{AIS,f}$ is the total conductance for length f. When substituting Equation 2 or 3 in Equation 4 we obtain the same result

$G_{AIS,f}=g_{n}\pi d_{n}l_{n}$ (5)

Thus, only assuming constant conductance density or constant area cannot reproduce the experimental results. However, when using both assumptions we get

$G_{AIS,f}=g_{n}\pi d_{n}\frac{l_{n}^{2}}{l_{f}}$ (5)

This assumption provides an inversely proportional relationship between the total final conductance of the AIS and its length. Simulations with this manipulation reproduce the experimental results, showing high intrinsic excitability for sound conditions, and low excitability for deaf and normal condition (Figure S2E). We repeated the simulations by co-varying the hillock. This shows that the hillock contributes to provide a graded transition between the low excitability responses in the normal and deaf conditions, and the high excitability observed in the sound condition (Figure S2F). It is important to note that our simulations also quantitatively reproduce the excitability response curves from the experiments (see the results for the number of spikes obtained at 200 pA in the description of Figure 7F). An experimental prediction is that as the length of the AIS changes under sound or deaf manipulations the diameter of this segment will vary.

Our models also show the change in the excitability under the different auditory manipulations in the number and sustainability of spiking (Figure S3A and S3B). Furthermore, the results also show that the spike amplitude is larger in the sound vs the normal condition (Figure S3C). Taken together, our simulations support the hypothesis that changes in the structure of the AIS are accompanied with changes in excitability.


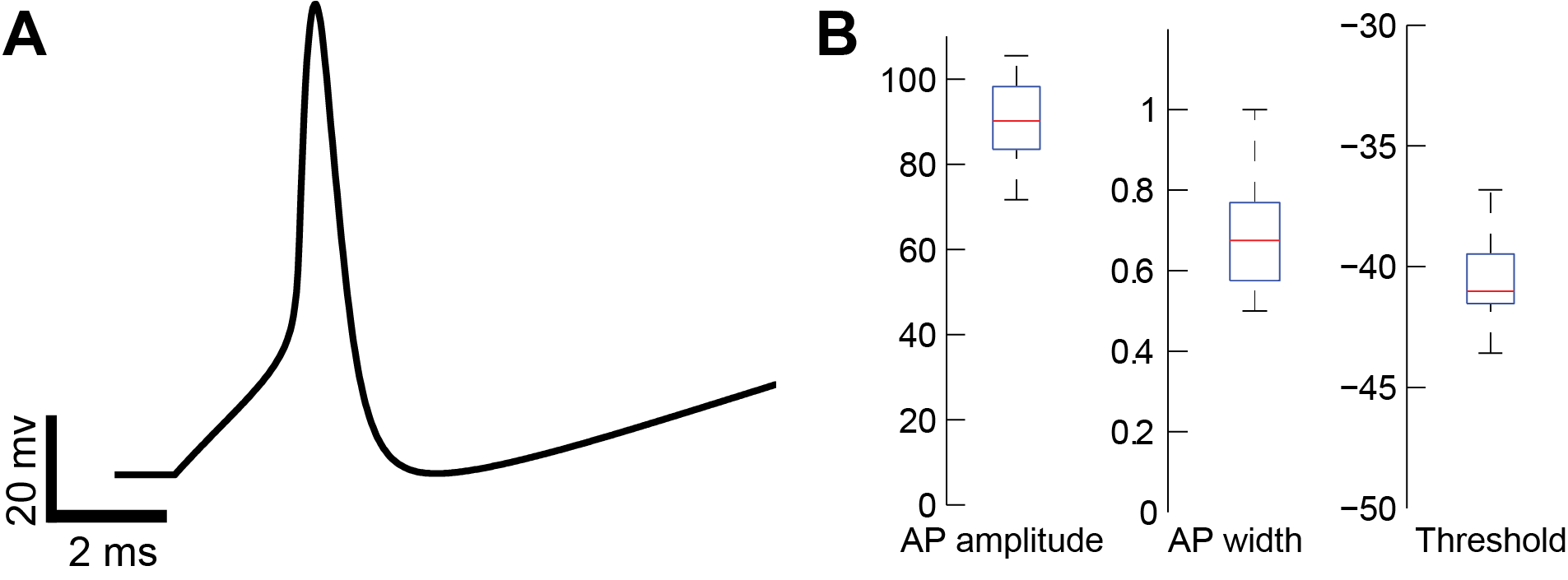


Figure 1. The MNTB model reproduces the action potential properties of the high frequency neurons. (A) example of an action potential in the model in response to a constant 200 pA stimulation. (B) Action potential amplitude, width at half-amplitude, and threshold for all the action potential obtained after delivering to the model a series of constant current steps from 0-300 pA. See text for parameters of the normal condition model.


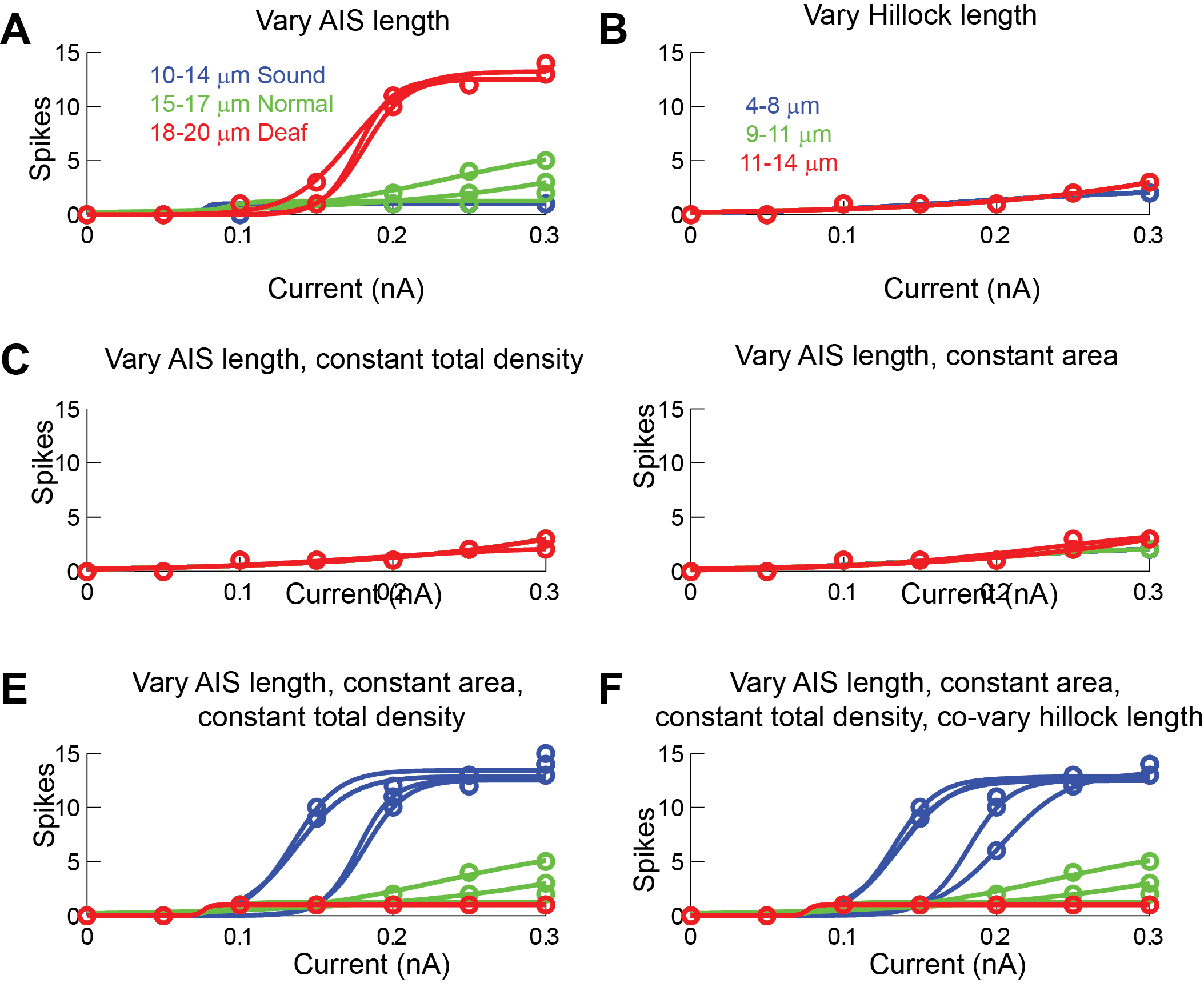


Figure 2. Reproducing the excitability changes in MNTB to different noise environments with a compartmental model. Number of spikes as a function of constant input current when varying the length of the (A) axonal initial segment (AIS) or (B) the hillock. In both cases the active properties remained fixed. (C) varying the AIS length while keeping the total conductance in the normal condition fixed. (D) varying the AIS length while keeping the area of the segment identical to the normal condition. (E) varying the AIS length while keeping the area and conductance identical to the normal condition. (F) same as E but co-varying the length of the hillock. See text for details of the model.


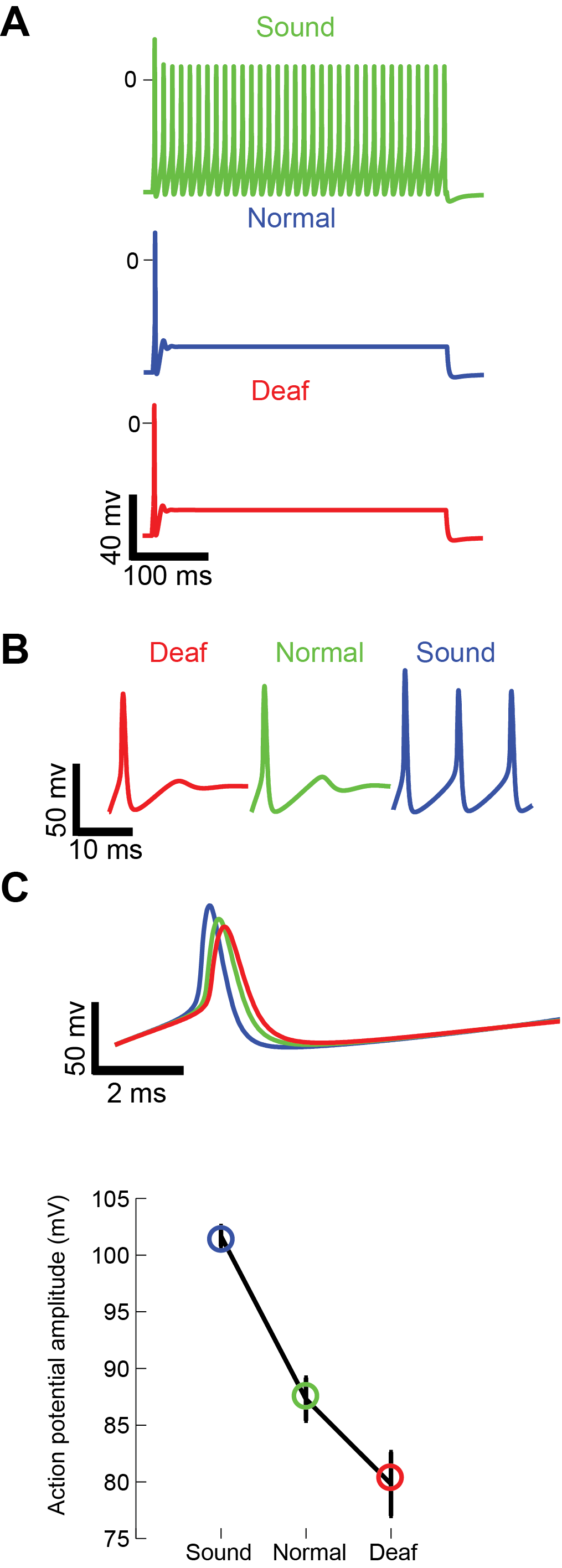


Figure 3. The MNTB model reproduces the spike properties measured in the deaf and sound manipulations. (A) sample traces of the model when stimulated by 200 pA of constant current with parameters that correspond to the sound (AIS 12 µm, hillock 6 µm), normal (AIS 16 µm, hillock 11 µm), and deaf (AIS 19 µm, hillock 13 µm). The conductances of the sodium and potassium channels in the AIS were modified as described in the text.

1. Leao RN, Leao RM, Da Costa LF, Rock Levinson S, Walmsley B. A novel role for MNTB neuron dendrites in regulating action potential amplitude and cell excitability during repetitive firing. European Journal of Neuroscience. 2008;27(12):3095-108.

2. Wang L-Y, Gan L, Perney TM, Schwartz I, Kaczmarek LK. Activation of Kv3. 1 channels in neuronal spine-like structures may induce local potassium ion depletion. Proceedings of the National Academy of Sciences. 1998;95(4):1882-7.

3. Yang Z, Santamaria F. Purkinje cell intrinsic excitability increases after synaptic long term depression. Journal of neurophysiology. 2016;116(3):1208-17.
